# Phylogeography of 912 Cherry Accessions Insight into Independent Origins of Fruiting Cherries and Domestication Footprints of Cultivated Chinese Cherry (*Prunus pseudocerasus* Lindl.)

**DOI:** 10.3390/plants12122258

**Published:** 2023-06-09

**Authors:** Tao Chen, Qing Chen, Jing Zhang, Yan Wang, Hao Wang, Yong Zhang, Ya Luo, Haoru Tang, Xiaorong Wang

**Affiliations:** 1 College of Horticulture, Sichuan Agricultural University, Chengdu 611130, China; chentao293@163.com (T.C.); supnovel@sicau.edu.cn (Q.C.); 71281@sicau.edu.cn (J.Z.); wangyanwxy@sicau.edu.cn (Y.W.); wh2sky@163.com (H.W.); zhyong@sicau.edu.cn (Y.Z.); luoya945@sicau.edu.cn (Y.L.); htang@sicau.edu.cn (H.T.); 2 College of Life Science, Sichuan Agricultural University, Ya’an 625014, China; 3 Institute of Pomology and Olericulture, Sichuan Agricultural University, Chengdu 611130, China

**Keywords:** subgenus *Cerasus*, Chinese cherry (*Prunus pseudocerasus* Lindl.), genetic divergence, independent origins, domestication, phylogeography

## Abstract

The subgenus *Cerasus* (Rosaceae) contain numerous fruit trees and ornamentals with high economic values. The origin and genetic divergence among various types of fruiting cherries always remain a perplexing issue. We employed three plastom fragments and ITS sequence matrices derived from 912 cherry accessions to elucidate the phylogeographic structure and genetic relationship among fruiting cherries, as well as the origin and domestication of cultivated Chinese cherry. The integration of haplotype genealogies, Approximate Bayesian computation (ABC) approach and estimation of genetic differentiation within and between different groups and lineages has facilitated the resolution of several previously unresolved questions. Firstly, distant phylogenetic relationships between *Cerasus* and *Microcerasus* accessions, as indicated by both nuclear and chloroplast data, suggested independent origins and evolution for these two taxa. Moreover, two distinct geographic origin centers (Europe and China) have been confirmed, with significant phylogeographic signals and high genetic differentiation observed between cherries from these regions. This may be attributed to long-term geographic isolation caused by Himalaya-Hengduan Mountains. Our phylogeographic analyses and ABC analysis suggested that cherries inhabiting in China may have undergone multiple hybridization events during the glacial refugia of the eastern edge and southern Himalaya-Hengduan Mountains, followed by rapid radiation throughout their current habitats during interglacial period. The discrepancy between nuclear and chloroplast data may be attributed to hybridization events and incomplete lineage sorting. Furthermore, we speculated that the domesticated Chinese cherries were derived from wild accessions in Longmenshan Fault Zones approximately 2600 years ago. We have also traced the domestication processes and dispersal routes of cultivated Chinese cherries.

## 1. Introduction

Cherry is an economically important group, belonging to the subgenus *Cerasus* of the family Rosaceae [1]. Cherry species have a wide distribution across Europe, North America, West and East Asia [1,2]. Majority of them (over 50 species) are predominantly distributed in Eastern Asia with a particular concentration in the mountainous regions of Southwest China (~45 species) [1,2]. These areas mainly encompass the eastern Himalaya-Hengduan Mountains (HHM) and adjacent regions, such as Qinling Mountains (QLM), Longmenshan Fault Zones (LFZ), and Yungui Plateau (YGP) [3]. As perennial woody trees with a long juvenile phase lasting from three to six years, cherry species exhibit multiple reproductive strategies, including both sexual breeding systems (e.g., self-fertilizing and out-crossing) and asexual modes of propagation (e.g., rooting suckers and scion grafting). In the field, they are frequently dispersed by insects, birds, animals and rivers. The combination of frequent geological activities in their habitats and diverse reproductive and dispersal strategies has resulted in a complex demographic history.

**Figure 1:**
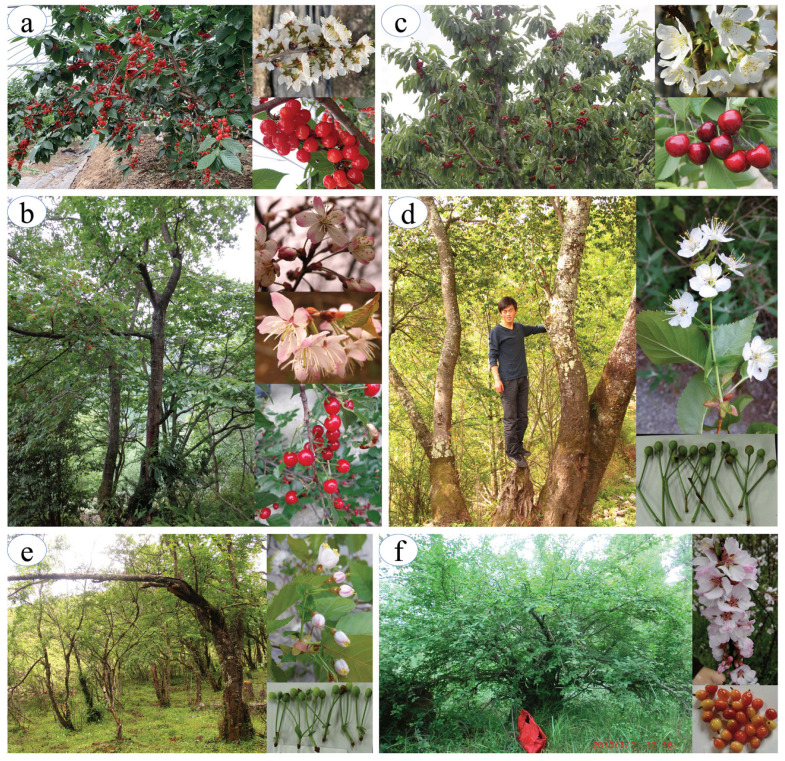
Characteristic members of the subgenus *Cerasus* and *Microcerasus* in their habitats and traits of their flowers and fruits. (**a**) Cultivated Chinese cherry (CC, *P. pseudocerasus*); (**b**) Wild Chinese cherry (WC, *P. pseudocerasus*); (**c**) European sweet cherry (EC, *P. avium*); (**d**,**e**) *Cerasus* relatives (RC, ~30 species and taxa); and (**f**) Dwarf cherry from subgenus *Microcerasus* (MC, *P. tomentosa*).

Cherry species comprise a diverse range of fruit trees (*P. pseudocerasus*, *P. avium*, *P. cerasus*, *P. tomentosa*, other species in subgenus *Microcerasus* etc., fruiting cherry) and ornamentals (*P. yedoensis*, *P. serrulata*, *P. campanulata*, *P. cerasoides* etc., flowering cherry), both of which have high economic and aesthetic values [1] (Figure 1). In general, the commercial fruit production relies on four major cultivar groups: sweet cherry (*P. avium*), sour cherry (*P. cerasus*), Chinese cherry (*P. pseudocerasus*) and dwarf cherry (*P. tomentosa*) [4]. Among the four types, sweet cherry and sour cherry have been utilized in Europe and Middle East for centuries. Over the past few decades, they have expanded to regions worldwide, including China after commercial introduction. In contrast, the Chinese cherry and dwarf cherry have a locally distribution range from northeast (Haiyang, Shandong province; E: 121°07.591′ N: 36°52.515′) to southwest of the nation (Kaiyuan, Yunnan province; E: 103°16.236′ N: 23°44.084′) [1,2,5].

Despite the widespread distribution and diverse species of subgenus *Cerasus*, there remains limited understanding regarding the origin and genetic relationship among various cultivated species, particularly with respect to Chinese cherry and sweet cherry. Whether all these distinct cultivated types have originated from a single or multiple location(s) is still unclear. In one scenario, it is plausible that the most recent wild common ancestor in China gave rise to geographically isolated lineages. China was once considered as a primary center of agriculture origins [6,7,8]. The earliest archaeological evidence of crop domestication in this region dates back to approximately 7000–10,000 B.C., and includes early indications of domesticated melon, cucumber, almond and mango [8]. In addition to its relatives, the peach was originally proposed to have been domesticated in China approximately 4000–5000 years ago with subsequent dispersion into Europe from Persia during the final centuries B.C. [9,10]. The domestication of cultivated apple (*Malus domestica*) has also been traced back to Northwest China, specifically near the Tianshan mountain range, along the Silk Route that spans from Asia to Western regions [11,12]. Furthermore, China boasts a rich array of cherry species (about 45 varieties of production), making it one of the centers of diversity [1,13]. The alternative hypothesis posits that Chinese cherry and sweet cherry arose from distinct domestication events, indicating at least two separate origins. The current sweet cherry is believed to have been domesticated from its wild ancestor ‘Mazzard’ around 4000–5000 years ago [14,15], with evidence suggesting a single domestication event. While Chinese cherry originated in China, their cultivation history can be traced back to 3000–4000 years. During the Xia and Shang dynasty (2070–1046 B.C.), the fruits of Chinese cherry were used as sacred objects [1]. Similar instances of independent domestication events in Europe and Asia for fruit crops have also been documented pears [16] and apricots [17]. However, the resolution of these studies is insufficient to unambiguously pinpoint the geographic region(s) associated with the origin of cultivated cherry.

Many molecular investigations have been conducted to explore the genetic diversity, population structure and phylogenetic relationships of cherry species. These studies have revealed that the evolutionary history of cherry species is highly complex and involves multiple hybridization events [18,19,20,21,22,23]. Nevertheless, the detailed demographic history of cherry species remains poorly investigated. Limited sample size and species in molecular studies pose challenges to providing effective information on the origin, genetic relationship and domestication of the economically important cultivated cherries.

Recently, the utilization of nuclear and chloroplast DNA sequences has played a crucial roles in analyzing the phylogeography and population genetics of plant crops [24]. Previous research has also confirmed the effectiveness of nuclear ITS (ITS1 + 5.8S + ITS2) sequences in investigating the population structure and phylogeography of cherry species [25]. Furthermore, chloroplast DNA markers, such as *matk* and *ndhF* gene sequences, intergenic spacer regions of *trn*Q-5′*rps*16, *trn*L-*trn*F and *atpB-rbcL* as well as the introns of and *rpl*16 and *rps*16 [25,26,27,28], have been effectively utilized to investigate the genetic relationship among cherry species or their relatives.

In this study, we selected one nuclear ITS and three chloroplast DNA markers (*rpl*16, *trn*L-*trn*F and *trn*Q-*rps16*) to conduct comprehensive phylogeographic analyses on 912 cherry accessions from 96 populations in 88 towns across 12 provinces of China. The accessions included 535 cultivated and wild Chinese cherries, 120 European cherry accessions, 174 accessions from other subgenus *Cerasus* taxa, and 79 accessions from the subgenus *Microcerasus* (Supplementary Figure S1, Table S1). Our sampling comprehensively covers the geographic distributions of Chinses cherry and its relatives in China, with the objective of (i) investigating the phylogeographic structure of these taxa, (ii) elucidating the genetic relationship among economically important fruiting cherry species, (iii) verifying the origin and domestication history of cultivated Chinese cherry.

## 2. Results

### 2.1. Sequence Characteristics

The complete concerted evolution of ITS has been well-documented, even in some allotetraploid taxa, resulting in intra-individual polymorphism been considered the exception [29]. However, some studies have identified its occurrence in various taxa including non-hybrid diploids and allopolyploids [30,31]. Fortunately, the present study has yielded encouraging results. Only two individuals showed a double-peak in their chromatograms when the ITS regions were amplified and directly sequenced. The lack of inter-individual ITS polymorphism suggests the potential application of ITS region in elucidating the phylogeographic pattern and demographic history of these species. 

Bulleted lists of all samples (Supplementary Table S1) are presented as follows: After eliminating low-quality sequences, a total of 906 ITS sequences were obtained, with lengths ranging from 700 to 709 base pairs (bp). The final ITS aligned dataset comprised 710 nucleotides, with ITS1 consisting of 227 bp, 5.8S rDNA comprising 207 bp, and ITS2 containing 276 bp. The GC content of the consensus sequence was 58.7%. A total of 121 polymorphic sites were detected, out of which 89 were phylogenetically informative. Sixty-nine haplotypes (H1–H69) were identified by polymorphic sites. Total haplotype and nucleotide diversity indexes were characterized by h = 0.7815 and π = 0.1416.

Following preliminary testing, three chloroplast DNA sequences (*rpl*16, *trn*L-*trn*F and *trn*Q-*rps*16) with high variation were selected for further analyses. The aligned DNA regions of *rpl*16, *trn*L-*trn*F and *trn*Q-*rps*16 were 1255 bp, 1048 bp and 1098 bp in length, respectively. Since no significant heterogeneity was detected by the partition homogeneity test, the sequences from the three regions were merged into a single cpDNA dataset consisting 3401 bp in length. After gap removal, the final concatenated cpDNA dataset consisted of 2313 bp with a GC content of 30.7%. By treating the indels as substitutions, we identified a total of 145 polymorphic sites and 107 parsimony-informative sites. A total of 113 haplotypes were identified, with corresponding haplotype and nucleotide diversity indexes of h = 0.7702 and π = 0.0824, respectively.

### 2.2. Haplotype Genealogy

We constructed four phylogenetic trees based on 69 ITS haplotypes and 113 chloroplast haplotypes, using both Maximus likelihood (ML) and Bayesian inference (BI) methods. The ITS datasets were fitted with the best-fit GTR model, while the chloroplast haplotypes were modeled using the best-fit GTR + G model. The BI and ML trees generated with the same dataset exhibited the highly congruent topologies. Therefore, we have only presented the BI tree for each dataset due to its more robust statistical supports. 

**Figure 2:**
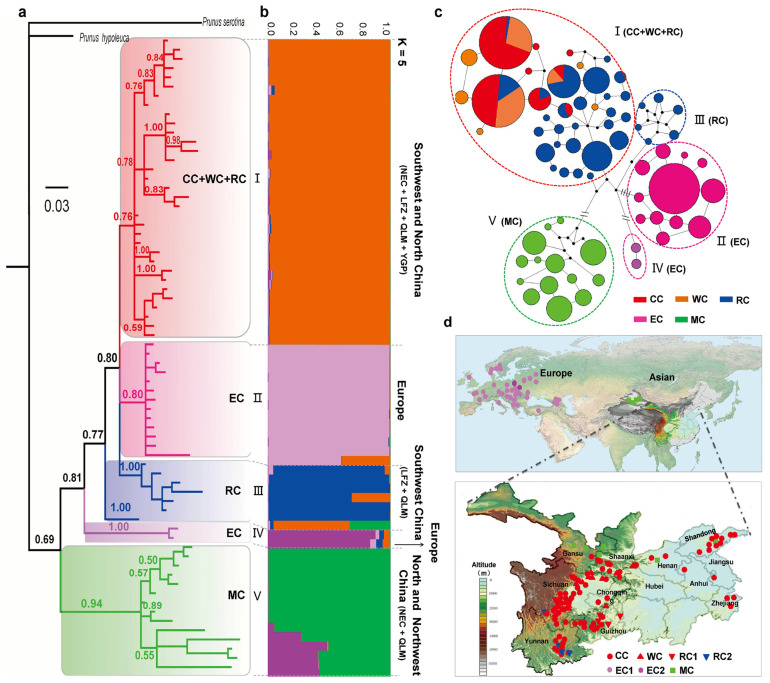
Genealogy and population genetics analysis of ITS haplotypes for 906 cherry accessions. (**a**) The Bayesian inference (BI) tree of 69 ITS haplotypes rooted by *P. serotina* and *P. hypoleuca*. The Bayesian posterior probability values ≥0.50 were displayed along the branches. (**b**) Model-based genetic structure of 69 ITS haplotypes under the *K* = 5. (**c**) A parsimonious median-joining network of ITS haplotypes. Node sizes are proportionate to haplotype frequencies within the dataset. Colors within the nodes correspond to the five groups in the key on the right, with black nodes representing inferred intermediate haplotypes. Double slashes between nodes indicate more than eight nucleotide changes. Dotted circles on the network delineate the five lineages identified through Bayesian Analysis Population Structure (BAPS) analysis. (**d**) Geographic locations of the five major lineages (I–V) were determined based on ITS sequence data. Symbols with different colors correspond to the five lineages in the legend.

The ITS haplotype phylogenetic tree was classified into five well-supported lineages (designated as I–V) (Figure 2a). The same five lineages were also identified through model-based STRUCTURE analysis, with an optimal number of *K* = 5 for 69 haplotypes (Figure 2b). These findings were further corroborated by the Median-joining network and BAPS analysis for the same set of 69 haplotypes (Figure 2c). Among the five lineages, lineage I comprises all CC and WC accessions as well as most of the RC accessions from NEC, QLM, LFZ and YGP; whereas lineage III consists of the remaining RC accessions from the LFZ and YGP. All studied EC accessions (*P. avium*, *P. cerasus* and their inter- and intra-specific hybrids) were present in lineage II, with the exception of *P. mahaleb* accessions which formed a distinct lineage IV. The basal lineage V comprises all MC accessions, including *P. tianshanica*, *P. tomentosa*, *P. humilis* and *P. glandulosa*. In general, *Microcerasus* accessions are significantly genetically distinct from those of *Cerasus*. European cherries exhibit relatively distant genetic relationships with their Chinese counterpart. 

**Figure 3:**
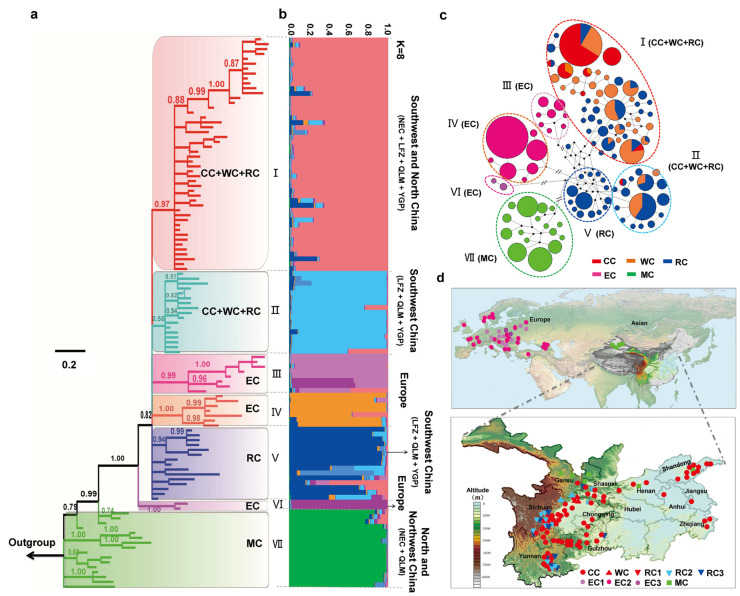
Genealogy and population genetics analysis of chloroplast haplotypes for 912 cherry accessions. (**a**) The Bayesian inference (BI) tree of 113 chloroplast haplotypes rooted by *P. kansuensis*, *P*. *persica* and *P*. *mume*. The Bayesian posterior probability values ≥0.50 were displayed along the branches. (**b**) Model-based genetic structure of 113 haplotypes under the *K* = 8. (**c**) A parsimonious median-joining network of chloroplast haplotypes. Node sizes are proportionate to haplotype frequencies within the dataset. Colors within the nodes correspond to the five groups in the key on the right, with black nodes representing inferred intermediate haplotypes. Double slashes between nodes indicate more than eight nucleotide changes. Dotted circles on the network delineate the seven lineages identified through Bayesian Analysis Population Structure (BAPS) analysis. (**d**) Geographic locations of the seven major lineages (I–VII) were determined based on chloroplast sequence data. Symbols with different colors correspond to the seven lineages in the legend.

Chloroplast phylogenetic analysis yielded seven distinct lineages (I–VII) with robust support values (Figure 3a), which was corroborated by our STRUCTURE analyses (Figure 3b), Median-joining network and BAPS analysis (Figure 3c). Consistent with the results of ITS haplotypes, all MC accessions were assigned to the basal lineage (VII), which was significantly distinct from *Cerasus* accessions. The EC accessions were classified into three distinct lineages (III, IV and VI) that exhibited relatively genetic divergence from other Chinese native cherries. Lineage III primarily comprised the hybrids of European cherries, while lineage IV corresponds to the majority of European sweet cherry cultivars. *P. mahaleb* accessions were also clustered into a distinct lineage (VI) separate from other European cherries. Moreover, the majority of CC, WC and RC accessions from NEC, QLM, LFZ and YGP were classified into lineage I. A few CC and WC accessions from LFZ and QLM were combined with RC accessions from LFZ, QLM and YGP in lineage II. The remaining accessions of LFZ, QLM and YGP were grouped into lineage V.

### 2.3. Genetic Diversity and Demographic History

Molecular diversity indices were calculated for various groups (CC, WC, RC, EC and MC) as well as lineages defined from haplotype analyses (Figure 2 and Figure 3) based on both ITS and chloroplast sequences (Table 1). Among the five groups, RC (ITS/cp: h = 0.8730/0.9279, π = 0.0447/0.0436) and MC (h = 0.8916/0.8559, π = 0.0837/0.0435) exhibited the highest levels of genetic diversity and nucleotide polymorphism in both nuclear and chloroplast data (Table 1), while CC (h = 0.5378/0.1440, π = 0.0062/0.0167) and WC (h = 0.5057/0.6528, π = 0.0076/0.0275) displayed the most minimal levels among all groups tested (Table 1). Among different lineages, the gene and nucleotide diversity of lineages III (part of RC) (h = 0.9333/0.9279, π = 0.0413/0.0436) and V (MC) (h = 0.8916/0.8576, π = 0.0837/0.0432) were significantly higher than those of other lineages (Table 1). The lineage I (CC + WC + RC) showed the lowest gene diversity (h = 0.6465/0.4217) for both ITS and chloroplast sequences (Table 1). In contrast, lineages IV (EC) (ITS: π = 0.0055) and VI (EC) (cp: π = 0.0046) exhibited the lowest nucleotide diversity in ITS and chloroplast sequences, respectively (Table 1).

Neutrality tests and mismatch distributions analysis (MDA) were performed on both ITS and chloroplast sequences (Table 1). For ITS sequences, significantly negative Tajima’s *D* (−1.5179, *p* = 0.0320) value were exclusively detected in group EC, and significantly negative Fu’s *F*s were observed in lineages I (CC + WC + RC) (−14.5974, *p* = 0.0060) and II (EC) (−14.7058, *p* = 0.0030) (Table 1). Unimodal mismatch distribution curves with significant *P*_Rag_ and *P*_SSD_ were detected in groups CC, WC and EC as well as lineages I (CC + WC + RC) and II (EC), while multimodal patterns were observed in the remaining groups and lineages (Supplementary Figure S2). For chloroplast data, both group CC (−2.1891, *p* = 0.000; −6.1829, *p* = 0.0020) and lineage II (CC + WC + RC) (−1.8758, P = 0.0080; −10.5326, *p* = 0.0000) exhibited significantly negative Tajima’s D and Fu’s *F*s values as well as unimodal mismatch distribution curves (Table 1, Supplementary Figure S3). The lineage I (CC + WC + RC) (−26.1216, *p* = 0.0000) exhibited significantly negative Fu’s *Fs* value but multimodal patterns (Table 1, Supplementary Figure S3). These results indicated that CC, WC and EC might experience recent population expansion.

The AMOVA analyses were performed to examine the genetic differentiation among groups and lineages. In both ITS and chloroplast data, the majority of total variation (57.92–84.02%) was found among different groups and lineages, with a smaller proportion detected among populations within groups and within lineages (10.75–18.63%), while an even smaller percentage was observed within populations (5.24–26.04%) (Table 2). Meanwhile, remarkably high *F*_CT_ values were observed among groups (ITS/cp: 0.7196/0.5792) and lineages (ITS/cp: 0.8402/0.7506) (Table 2). The results collectively suggest a significant genetic divergency among various groups and lineages. We further computed the pairwise genetic differentiation values among groups and lineages. In both ITS and cpDNA sequences, remarkably high pairwise *Fst* values (*Fst* > 0.600) were also observed among almost all lineages (Supplementary Tables S2 and S3), between MC and other groups (Supplementary Figure S4a), and between EC and Chinese native groups (CC, WC and RC) (Supplementary Figure S4a). The lowest *Fst* value was 0.0157 (*p* < 0.05) between groups CC and WC for the ITS data and was 0.1681 (*p* < 0.01) between groups WC and RC for the chloroplast data (Supplementary Figure S4a).

**Figure 4:**
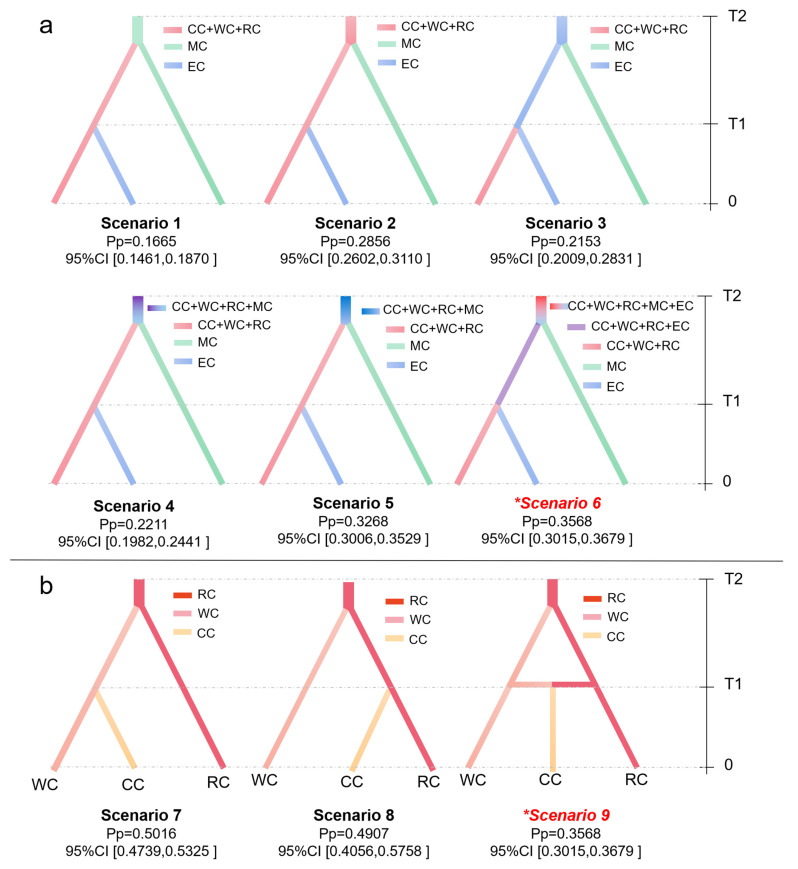
Nine potential scenarios of divergence among various cherry groups compared using approximate Bayesian computation (ABC) analysis. (**a**) Six potential scenarios of divergence among cherries from different taxa and geographic regions. (**b**) The three potential scenarios of divergence among different cherries native to China. Bars in the right side indicate the timescale. T1: the time of the second divergence event; T2: the time of the first divergence even (T2 > T1 > 0; 0 = present). For each scenario, the corresponding posterior probability and 95% confidence intervals (CI) are presented below. The most probable scenario (*) was highlighted in red italics, which was selected based on the highest posterior probability and the mean values of Types I–II error lower than 20%.

The ABC analysis was performed on all datasets to infer the divergency history of cultivated cherries. Six possible scenarios were designed for three groups (Figure 4a), and moderated probabilities were detected in each scenario with the posterior probability range from 0.1665 (scenario 1, 95% CI: 0.1461–0.1879) to 0.3568 (scenario 6, 95% CI: 0.3015–0.3679) (Figure 4a). Scenario 6 had the highest posterior probability and described the best fit hypothesis for worldwide cherries (Figure 4a). The type I error and mean type II error for scenario 6 were 0.130 and 0.053 (<20%), respectively, indicating a high level of confidence in our optimal hypothesis choices. The scenario 6 portrays the emergence of all the cherry species from a common, uncertain ancestor (mixed group CC + WC + RC + EC + MC). Taxon MC firstly diverged as an independent lineage at 3.52 Ma (Million years ago) (95% CI: 0.89–6.50 Ma), followed by the origin of group EC from a mixed ancestral group (CC + WC + RC + EC) at 3.52 Ma (95% CI: 0.89–6.50 Ma). The current effective population sizes of mixed Chinese cherry group (CC + WC + RC) (N1 = 43,900, 95% CI: 14,200–87,100) and MC group (N3 = 37,300, 95% CI: 31,800–56,500) were significantly higher than that of EC group (N2 = 1610, 95% CI: 437–6760) (Supplementary Table S4).

### 2.4. Phylogeographic Structure of Chinese Cherry

**Figure 5:**
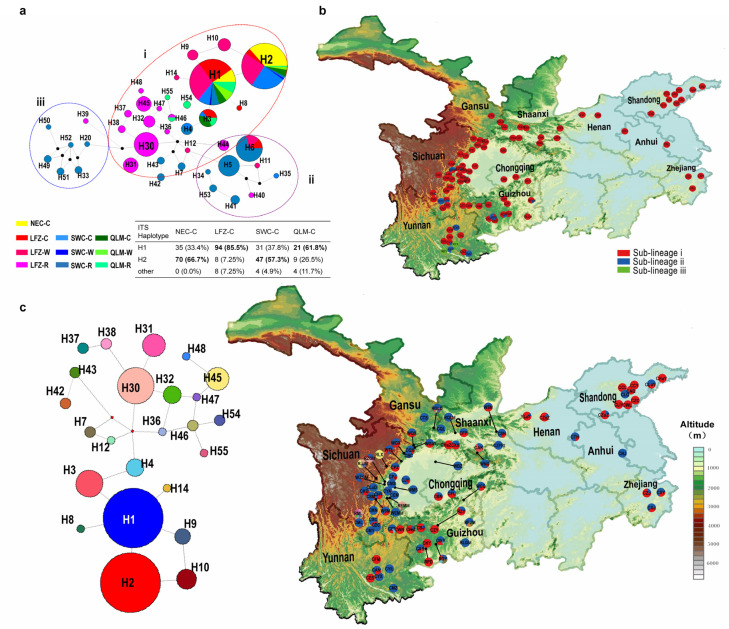
Phylogeographic analysis of Chinese cherry (CC and WC) and its relatives (RC) based on ITS sequences. (**a**) ITS Haplotype network. Node sizes are proportionate to haplotype frequencies in the dataset. Colors within the nodes represent ten datasets correspond to those in Supplementary Table S1. Dotted circles demarcate the three sub-lineages (i, ii and iii) based on Bayesian Analysis Population Structure (BAPS) analysis. The haplotype composition of cultivated Chinese cherry is presented as a percentage across four geographic regions. (**b**) Geographic locations of the three sub-lineages. Symbols with different color correspond to the sub-lineages in the legend. Population codes are identified in Supplementary Table S1. (**c**) Distribution of the ITS haplotypes within sub-lineage i. Population codes are listed in Supplementary Table S1. The left side panel displays the median-joining network of sub-lineage i.

In order to elucidate the origin of cultivated Chinese cherry, a phylogeographic analysis was further conducted on Chinese cherry and its relatives (groups CC, WC and RC). Forty ITS haplotypes were identified in the three groups, which could be divided into three sub-lineages (i, ii and iii) based on haplotype network using BAPS (Figure 5a). The sub-lineage i encompasses all CC, WC and portion of RC accessions that span across the majority of cherry distribution regions in China (Figure 5b). Sub-lineages ii and iii comprise the majority of RC accessions from southwestern China (Figure 5a,b). Haplotype H30 is the central node of three sub-lineages, comprising RC accessions from populations EMM, ML and JJM in the LFZ region (Figure 5a,b, Supplementary Table S1). With further consideration of sub-lineage i, haplotypes H1 and H2 had the largest sample size, primarily consisting of cultivated Chinese cherry. H1 was predominantly found in cultivated Chinese cherry from LFZ and QLM regions (LFZ-C and QLM-C), with a proportion of 85.50% and 61.80%, respectively (Figure 5a). The H2 haplotype was comprised of cultivated accessions from NEC and YGP (NEC-C and YGP-C), which accounted for 66.70% and 57.30%, respectively (Figure 5a). The remaining surrounding haplotypes were predominantly associated with the RC group from LFZ and YGP regions (Figure 5a,c).

**Figure 6:**
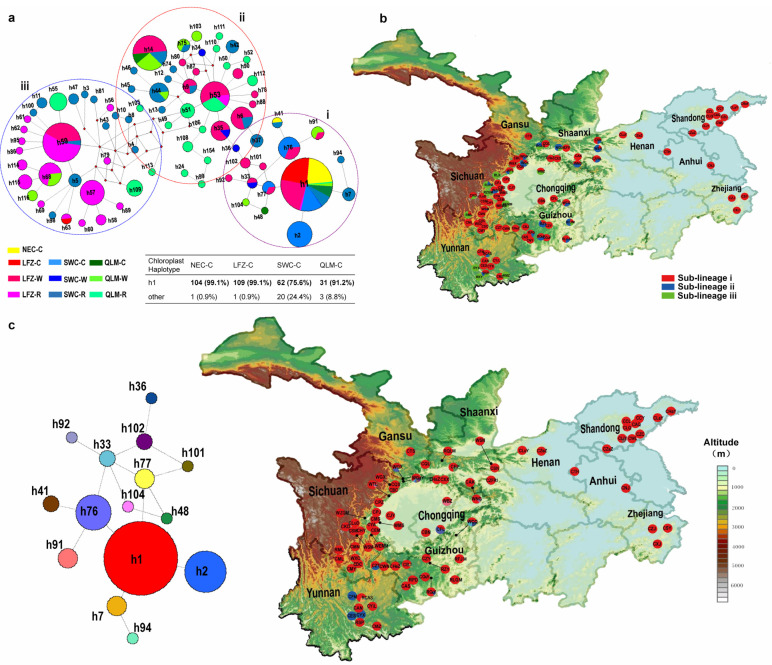
Phylogeographic analysis of Chinese cherry (CC and WC) and relatives (RC) based on chloroplast DNA sequences. (**a**) Chloroplast Haplotype network. Node sizes are proportionate to haplotype frequencies in the dataset. Colors within the nodes represent ten datasets correspond to those in Supplementary Table S1. Dotted circles demarcate the three sub-lineages (i, ii and iii) based on Bayesian Analysis Population Structure (BAPS) analysis. The haplotype composition of cultivated Chinese cherry is presented as a percentage across four geographic regions. (**b**) Geographic locations of the three sub-lineages. Symbols with different color correspond to the sub-lineages in the legend. Population codes are identified in Supplementary Table S1. (**c**) Distribution of the chloroplast haplotypes within sub-lineage i. Population codes are listed in Supplementary Table S1. The left side panel displays the median-joining network of sub-lineage i.

A total of 80 chloroplast haplotypes were identified in the CC, WC and RC groups, which were also classified into three sub-lineages (i, ii and iii) based on haplotype network and BAPS analysis (Figure 6a). Sub-lineage i was found to be present in all three groups, encompassing their entire distribution regions. Meanwhile, sub-lineages ii and iii were shared exclusively between the RC and WC group across LFZ, YGP and QLM regions (Figure 6a,b). According to coalescent theory, h53 is inferred as one of the oldest haplotypes, primarily covering the WC and RC accessions from the LFZ. Haplotype h59 was located at the central node of sub-lineage iii and predominantly consisted of the RC accessions from the LFZ region, representing the ancestral haplotype shape with a radiative evolutionary pattern (Figure 6a). Considering the sub-lineage i, haplotype h1 has the largest sample size (305) and is associated with predominant cultivated Chinese cherry. The WC group gave rise to the adjacent haplotypes (Figure 6a,c).

We calculated the genetic diversity indices and conducted neutrality tests and MDA for various geographic subgroups of CC, WC and RC groups as well as the identified sub-lineages (i–iii) (Table 3, Supplementary Figure S3). In the ITS data, LFZ-C (h = 0.2647, π = 0.0054), NEC-C (h = 0.4487, π = 0.0037) and QLM-W (h = 0.2540, π = 0.0021) exhibited low levels of genetic diversity, whereas LFZ-R (h = 0.8434, π = 0.0249) and YGP-R (h = 0.8536, π = 0.0540) displayed high genetic diversity (Table 3). In terms of chloroplast data, the genetic diversity observed in NEC-C, LFZ-C, YGP-C and QLM-C was significantly lower than that found within the six geographic subgroups of WC and RC (Table 3). Among different geographic regions, LFZ-C and YGP-C showed significantly negative Tajima’s *D* values in ITS sequences. In chloroplast data, NEC-C and LFZ-C exhibited significantly negative Tajima’s *D* values, while YGP-R had significantly negative Fu’s *Fs* value. Considering the sub-lineages, low genetic diversity, significantly negative Tajima’s *D* and Fu’s *F*s values as well as unimodal MDA curves were detected in sub-lineage i for both ITS and chloroplast data (Table 3, Supplementary Figures S2 and S3).

In our AMOVA analyses, minimal levels of total variation were detected among the three groups and among different geographic subgroups within each group (Table 2). The percentage of variation ranged from 18.41% (RC) to 26.73% (WC) for ITS, and from 2.69% (WC) to 30.05% (among three groups) for chloroplast data (Table 2). Furthermore, most geographic subgroups of CC showed low pairwise F*st* values (F*st* < 0.2500) in both ITS and chloroplast data. However, moderate F*st* values were observed between NEC-C and LFZ-C (F*st* = 0.3930, *p* < 0.01) as well as between QLM-C and LFZ-C (F*st* = 0.2534, *p* < 0.01) in the ITS data (Supplementary Figure S4b). Low level of genetic differentiation (F*st* = 0.0025–0.2103) was observed among all geographic subgroups of CC and WC in ITS data. However, low levels of genetic differentiation were only observed between LFZ-W and four subgroups of CC (*Fst* = 0.0894–0.2016), while the four cultivated subgroups exhibited significant genetic differentiation from YGP-W (*Fst* = 0.3691–0.8558) and QLM-W (F*st* = 0.3117–0.6204). Furthermore, the majority of the geographic subgroups within WC and RC exhibit low or moderate levels of genetic differentiation (ITS/cp: *Fst* = 0.0203–0.3065/0.1737–0.4754) with one another (Supplementary Figure S4b).

Three hypothetical scenarios (scenario 7–9) were formulated to infer the domestication history of Chinese cherry (Figure 4). The result suggests that scenario 9 provides the best fit to our data, with the highest posterior probability of 0.5853. This scenario indicates that WC group diverged from the RC group approximately 34,200 years ago (95% CI: 131,00–97,600), and subsequently experienced frequent gene flow between RC and WC. The cultivated Chinese cherry (CC group) split off at about 2660 years ago (95% CI: 1180–5540) (Supplementary Table S5). the RC group maintains the highest current effective population sizes of 37,500 (95% CI: 4950–82,800), followed by the WC group (5890, 95% CI: 1050–9740). The CC group has the lowest current effective population sizes of 1530 (95% CI: 1180–5550) (Supplementary Table S5).

## 3. Discussion

### 3.1. Independent Origins of Economically Cultivated Fruiting Cherry

Due to the parallel evolution of morphological traits, frequent intra- and inter-specific hybridization, and complex biogeographic histories [18,21,23,27], the exploration of the origin and evolution of cherry species poses a challenging task for researchers. In this study, we conducted an analysis of the nuclear and chloroplast DNA sequences from more than 900 cherry accessions. We reconstructed the parental and maternal phylogenetic networks to investigate the phylogeographic structure and demographic history of the samples.

Both nuclear and chloroplast data indicate distant phylogenetic relationships between subgenus *Cerasus* and *Microcerasus* accessions (Figure 3 and Figure 4). Previous molecular studies [18,27,32,33,34] indicated that *Microcerasus* was much genetically closer to the subgenus *Amygdalus*, *Armeniaca* and *Prunus* than to subgenus *Cerasus*. Although the precise genetic origin of subgenus *Microcerasus* could not be confirmed due to lack of members in homologous subgenus, our data suggests a relatively distinct evolutionary history between subgenus *Cerasus* and *Microcerasus* (Figure 2, Figure 3 and Figure 6). Meanwhile, the high level of genetic diversity and constant population size observed in *Microcerasus* (group and lineage of MC) (Table 1, Supplementary Figure S2 and Table S4) indicate that no genetic bottleneck occurred during their recent evolution. Similar conclusion has also been documented in previous study [35].

The haplotype genealogy, genetic diversity and demographic evolution indicate significant genetic divergence within the subgenus *Cerasus*. In both nuclear and chloroplast sequences, all European *P. mahaleb* accessions formed a basal clade in *Cerasus* (Figure 2a and Figure 4a), suggesting their early divergence and distinct evolutionary history from other *Cerasus* taxa. Previous studies have also reported the unique phylogenetic history of *P. mahaleb*, which is characterized by a distinctive evolutionary pattern and controversial classification [27]. While other European populations developed distinct paternal and maternal lineages that were separate from those found in China (Figure 2a, Figure 4a and Figure 6). These data suggest the presence of two distinct origin and evolutionary histories between European and Chinese cherries. Recent molecular dating based on whole genome-wide sequences [13] indicates that the divergence of European and eastern Asian cherries occurred during the early Pliocene, a period subsequent to the massive uplift of the Qinghai-Tibetan Plateau (QTP) and Himalaya-Hengduan Mountains (HHM). Significant topographic changes and climate shifts resulted in the rapid radiation and geographic isolation of plant species in this region [36,37]. Taking into account the hypothesis of independent origins of cherry species [18], it is postulated that the wild progenitors of these European cherries diverged from other *Cerasus* taxa in eastern and western Asia, and rapidly spread to Europe. Subsequently, the uplift of the QTP and HHM obstructed gene exchanges between European and eastern Asian cherries, which might have contributed to their clear genetic divergence and separated evolutionary histories. Our ABC ananlysis results have indicated that the European cherry (group EC) divergced from a mixed ancestral group (CC + WC + RC + EC) approximately 3.52 Ma (95% CI: 0.89–6.50 Ma), which lends further support to this hypothesis (Supplementary Table S4).

Multiple genetic lineages were observed in *Cerasus* (Figure 2 and Figure 3), indicating a complex evolutionary history. The incongruences between nuclear and chloroplast genealogies were observed, with *Cerasus* accessions being clustered into lineage I and II in nuclear data, and three distinct lineages (I, II and V) in chloroplast data (Figure 5 and Figure 6). Gene tree conflicts can arise from two primary factors, hybridization and incomplete lineage sorting. Recent genome-wide data indicate that there have been four potential instances of hybridization among cherry species in China [23]. Our field investigation has also found numerous cherry resources with overlapping distribution and intermediate morphological traits of multiple cherry species in LFZ, QLM and YGP [3]. Therefore, we consider that hybridization is the driving force behind the incongruence. Moreover, incomplete lineage sorting may also be a crucial cause in generating shallow-level phylogenies based on limited nuclear and chloroplast DNA markers [38,39,40,41], particularly when plant species have undergone recent rapid diversification and possess very large effective population (e.g., radiative evolution) [40]. This is also holds true for the case of partial cherry resources of China. Thus, we propose that the observed incongruences may be attributed to frequent hybridization events and incomplete lineage sorting.

Considering the four geographic regions, high genetic diversity was detected in wild resources from LFZ and YGP (LFZ-W, LFZ-R, YGP-W and YGP-R) (Table 3). The center nodes were also primarily consisted of these source populations, without discernible phylogeographic signals within them (Figure 5 and Figure 6). Meantime, in comparison to other regions, the high-altitude regions of LFZ and YGP harbored greater degree of species richness and intraspecific diversity with regard to cherry resources [3]. Based on these facts, it can be inferred that the high-altitude areas of LFZ and YGP (the eastern edge and southern regions of the HHM) serve as primary glacial refugia for cherry species, where frequent hybridization events could occur and subsequently lead to rapidly radiation throughout QLM, LFZ, YGP and other habitats during interglacial periods. This inference is further supported by the relatively low and moderate levels of genetic differentiation observed between the various geographic subgroups of WC and RC, as well as the ABC analysis (Figure 4, Supplementary Table S5). The eastern and southern regions of the HHM have also been proposed as glacial refugia for many other plant species [42,43,44,45,46,47].

Overall, our data suggests that the economically cultivated fruiting cherry species have independent origins. *P. tomentosa* from the subgenus *Microcerasus* exhibits high genetic differentiation from other fruiting cherries and evolved independently. The QTP and HHM may intensify genetic differentiation, resulting in the divergence of domestication between cultivated fruiting cherries in Europe (e.g., *P. avium* and *P. cerasus*) and China (e.g., *P. pseudocerasus*). In terms of chloroplast haplotype network, a noticeable divergence was observed between *P. avium* and most hybrid types among European fruiting cherries, while no significant difference was detected in nuclear haplotype network. Considering the maternal inheritance of chloroplast sequences in higher plants [48], it is possible that European fruiting cherries underwent separate evolutionary processes during speciation, followed by hybridization and gene introgression between different European lineages resulting in an admixed gene component. Concerted evolution of ITS sequence has been reported in the evolutionary process of Rosaceae fruit trees, such as, *Malus* [49] and *Crataegus* [50].

### 3.2. Geographical Origin and Domestication of Cultivated Chinese Cherry

As mentioned above, multiple instances of hybridization events might occur among cherry species in China. Under this scenario, chloroplast sequences are powerful tools for exploring the origin and domestication of plant species, owing to their maternal inheritance. In chloroplast data, the genetic differentiation between cultivated Chinese cherry (the four subgroups LFZ-C, YGP-C, QLM-C and NEC-C) and LFZ-W was much lower compared to other wild subgroups (QLM-W and YGP-W). This provides molecular evidence supporting our recent hypothesis [25,51] that all cultivated Chinese cherries likely originated in LFZ. Furthermore, in accordance with our recent investigation [51], the cultivated Chinese cherry is predominantly classified into two nuclear haplotypes (H1 and H2) (Figure 5a). Compared to YGP-C and QLM-C accessions, LFZ-C and NEC-C accessions exhibited relatively homogeneous gene pools (Figure 5 and Figure 6) and low level of genetic diversity (Table 3). Significant differences in fruit traits were also observed between LFZ-C and NEC-C accessions [52,53], indicating that LFZ and NEC may be the two separate major domestication sites of cultivated Chinese cherry.

Based on nuclear and chloroplast data, we traced the domestication processes and potential dispersal routes of cultivated Chinese cherry (*C. pseudocerasus*) The domestication and migration of woody fruit trees have been demonstrated to be closely associated with anthropogenic civilization, especially the revolutionary improvements and dissemination of agricultural techniques (e.g., cuttings, suckers and scion grafting) [12,51,54,55,56,57]. Herein, we hypothesize that the domestication of Chinese cherries from a mixed gene pool of its wild ancestor in LFZ occurred approximately 3000 years ago [51] (Figure 4, Supplementary Table S5). Subsequently, clonally propagated techniques (e.g., grafting) facilitated their dissemination to the ancient political and agricultural center NEC via gallery road of QLM, as well as to YGP along ancient trade road. Over the past millennia, divergent selections in fruit traits were achieved at the two major domestication sites (LFZ and NEC) due to distinct domestication preferences. This scenario also can be supported by morphological and molecular studies as well as fossils and writing records [5,25,51,52,53,58,59]. In addition, high *Fst* values (*Fst* = 0.4165–0.5494) were detected between the CC and RC, indicating few gene introgression between cultivated Chinese cherries and their close relatives from other *Cerasus* taxa. This is different from those of other Rosaceae fruit trees, such as apple [11,56,57] and pear [16].

## 4. Materials and Methods

### 4.1. Plant Materials

In this study, a total of 912 accessions (~30 species and varieties) were collected and classified into five groups: cultivated Chinese cherry (CC), wild Chinese cherry (WC), wild relatives (RC), European cherries (EC) and *Microcerasus* (MC). In subgenus *Cerasus*, the sample set consists of 535 accessions of Chinese cherry, 120 European cherry accessions and 174 accessions of other wild relatives. All 535 Chinese cherry specimens, comprising 331 cultivated (CC) and 204 wild individuals (WC), were systematically collected from a total of 70 natural populations (534 accessions) and Zhengzhou Fruit Research Institute, Chinese Academy of Agricultural Sciences (ZFRI) (1 accession). The European cherry (EC), including cultivars of *P. avium*, *P. cerasus*, *P. mahaleb* and their inter- and intra-specific hybrids, were collected from ZFRI in a professional manner. All the wild relatives (RC) were collected from 16 natural populations (155 accession) and Kunming Institute of Botany, Chinese Academy of Science (KIB, CAS) (19 accession) (Supplementary Figure S1, Table S1). In subgenus *Microcerasus*, a total of 79 accessions were collected (MC), comprising 48 individuals of *P. tomentosa* from 8 natural populations, 26 samples of *P. tianshanica* from 2 natural populations, and 3 and 2 specimens of *P. japonica* var. *salicifolia* and *P. japonica* var. *glandulosa* respectively from ZFRI (Supplementary Table S1). Four plausible geographic areas in China were designated as follow: North and East China (NEC), Longmenshan Fault Zones (LFZ), Yun-Gui Plateau (YGP) and Qinling Mountain (QLM). Within group CC, four subgroups were designed (NEC-C, LFZ-C, YGP-C and QLM-C); within group WC three subgroups were classified (LFZ-W, YGP-W and QLM-W); within group RC also three subgroups were identified (LFZ-R, YGP-R and QLM-R) (Supplementary Figure S1, Table S1). Additionally, *P. serotina*, *P. hypoleuca*, *P. kansuensis*, *P. persica* and *P. mume* were chosen as outgroups. 

### 4.2. DNA Isolation, PCR Amplification, and Sequencing

Total genomic DNA was extracted from silica-gel dried leaf tissues using a modified CTAB protocol [26]. In the initial testing phase, eight regions of the chloroplast genome (*matk* and *ndhF* gene; intergenic spacer of *trn*Q-5′*rps*16, *trn*L-*trn*F and *atpB-rbcL*; introns of *rpl*16 and *rps*16) were selected to examine their variation by using six cultivated and four wild *P. pseudocerasus* accessions. Subsequently, three regions of chloroplast genome (*trn*L-*trn*F, *trn*Q-*rps*16 and *rps*16 intron) exhibiting high variation were remained and utilized for genotyping all 912 accessions (Supplementary Table S6). In order to ensure the suitability of nuclear ITS fragments for species level phylogeographic analysis, we first checked their characterization with regards to intra-individual ITS polymorphism and pseudogenes, following the guidelines for handling ITS sequences [60] and drawing on our previous molecular study [25]. The PCR conditions were performed to the methods of Chen et al. [25]. PCR products were directly sequenced using the Big Dye Terminator Cycle Sequencing kit (Applied Biosystems, Foster City, CA, USA) on an ABI PRISM 3730 automatic DNA sequencer (Applied Biosystems, Foster City, CA, USA). All sequences have been deposited in the GenBank database and assigned accession numbers KX711711-KX711781.

### 4.3. Sequence Alignment

Raw sequences were carefully examined and refined using DNASTAR5.0 (DNASTAR Inc., Madison, WI, USA). The sequences from the three chloroplast regions and nuclear ITS fragment were aligned separately with CLUSTAL_X 1.81 [61] and manually adjusted in MEGA5.0 [62]. We combined the three chloroplast datasets into a super chloroplast dataset using Sequence Matrix 1.7.8 [63], based on the results of partition homogeneity test (PHT) [64] in PAUP 4.0 [65]. Finally, a comprehensive chloroplast dataset and nuclear dataset were obtained. The insertions or deletions (InDels) within the two datasets were treated as substitutions using the Fast-gap program [66].

### 4.4. Haplotype Genealogy Construction

The identification of Nuclear DNA (nrDNA) and Chloroplast DNA (cpDNA) haplotypes was performed separately for each datasets in DNAspV5 [67], with default settings applied. Haplotype networks were constructed using the median-joining method [68] in Network4.2.0.1 [69] and the statistical parsimony criteria in TCS1.2 [70]. Phylogenetic structure of nrDNA and cpDNA haplotypes were inferred using both the Bayesian inference (BI) and Maximus likelihood (ML) methods. The optimal models of nucleotide substitution were determined using Akaike Information Criterion (AIC) [71] in JModelTest 2 [72]. The BI analysis was conducted with the MrBayes 3.1.2 [73]. Two independent runs were carried out. For each run, Markov Chain Monte Carlo (MCMC) algorithm was performed for 3 × 10^6^ generations with one cold and three heated chains, starting with different random trees and sampling trees every 100 generations. The first 8000 generations were discarded as burn-in. The ML analysis was performed using IQ-TREE V.1.5.5 [74] with 1000 regular bootstrap replicates. All the dendrograms were edited and presented using FigTree v.1.4.4 (https://github.com/rambaut/figtree, accessed on 26 November 2018).

Bayesian Analysis of Population Structure version 5.4 (BAPS 5.4) was employed to deduce the genetic structure of each designated lineage based on haplotype data from both nrDNA and cpDNA. The BAPS method uses MCMC simulation to partition different populations or individuals into variable groups (*K*). Ten algorithm repetitions were performed for each value of *K* (1–20). The *K* value with highest marginal log (marginal likelihood) indicates the optimal number of groups that best fits the data. Meantime, the genetic structures within lineages were also detected using Structure 2.3 [75]. Twenty independent runs were performed for each value of *K* (1–10) using admixture model and correlated allele frequencies. The MCMC algorithm was run for 100,000 burn-in itertions followed by 100,000 additional generations. The mean membership coefficients (Q) matrix was calculated for each *K* by using the estimated Q-matrix from 20 runs of each *K* in CLUMPP software [76], and subsequently visualized using DISTRUCT [77]. The optimal number of genetic clusters (*K*) was determined using the method proposed by Evanno et al. [78] and implemented in the online program Structure Harvester (http://taylor0.biology.ucla.edu/structureHarvester/, accessed on 1 July 2014) [79].

### 4.5. Molecular Diversity and Population Structure

We categorized our specimens into five groups based on their taxonomic classification and geographic distribution, namely cultivated Chinese cherry (CC), wild Chinese cherry (WC), European cherries (EC), other members of the subgenus *Cerasus* (RC) and *Microcerasus* (MC) (Supplementary Table S1). Distinct genetic lineages were also assigned to different groups based on the results of haplotype networks. Molecular diversity indices for each group and lineage were calculated using ARLEQUIN 3.5 [80]. The variance components and significant levels of variations for each group were calculated through the implementation of Analysis of molecular variance (AMOVA). Genetic differentiation was assessed by pairwise *Fst* values. Both AMOVA and *Fst* analysis employed Tamura and Nei’s genetic distance [81] to account for heterogeneity of mutation rates.

### 4.6. Historical Demographic Analysis

Neutrality tests were utilized to identify potential historical population events. Tajima’s *D* [82] and Fu’s *F*s statistics [83] were calculated using ARLEQUIN 3.5 for various groups and lineages. Assuming neutrality, a population expansion often results in significantly negative values of Tajima’s *D* and Fu’s *F*s, indicating strong evidence for demographic growth. To further investigate the historical dynamics of both geographic and genetic groups, a mismatch distributions analysis (MDA) was performed using a sudden demographic expansion model with 5000 permutations in ARLEQUIN 3.5. Statistical significance was tested by *P*_Rag_ and *P*_SSD_, respectively. This analysis compared the observed frequencies of pairwise differences in haplotypes with those expected under a single sudden expansion model [84]. It is know that sudden population expansion leads to unimodal distribution [85].

In order to further investigate the historical demography of fruiting cherries, we employed an Approximate Bayesian Computation (ABC) algorithm using DIYABC v.2.1.0 software to establish the most probable scenarios for their domestication history. Based on the taxonomic classification, PCA and phylogenetic tree analysis, all the accessions were categorized into three groups: mix group (CC + WC + RC), group EC, and group MC. According to the significant divergence between *Microcerasus* and other species, as well as its base location in the phylogenetic tree, we first tested six potential scenarios of divergence to determine whether cultivated cherries originated from *Microcerasus*. Subsequently, we classified the mix group into CC, WC and RC categories and established three possible scenarios to estimate the probable domestication history of cultivated Chinese cherry (Figure 4). The Generalized Stepwise Mutation model (GSM) was selected for the combined four fragment sequence dataset in DIYABC v2.1.0. The mean mutation rate (µ) was sampled from a uniform distribution with default values of 10^−4^ and 10^−3^, consistent with those employed in the study of perennial woody trees [48]. A conservative estimate of a 4-years generation time was estimate based on our field observations to infer the demographic history of cherries. One million simulated datasets were generated for each scenario to facilitate subsequent analysis. A uniform prior probability was employed, and all summary statistics were chosen to generate a reference table. The most appropriate scenario for each hypothesis was determined by comparing the summary statistics of simulated and observed datasets. Using a logistic regression approach, the posterior probabilities for each competing scenario were estimated along with their 95% confidence intervals (CI). These estimates were based on the 1% of simulated datasets that most closely resembled to the observed dataset.

## 5. Conclusions

Phylogeographic analyses of 912 cherry accessions indicated distinct genetic differentiation and evolutionary process among cherry species, especially between those belong to the subgenus *Cerasus* and *Microcerasus*. The distinct genetic divergence and separate domestication processes observed between cultivated cherry species from Europe and those from China can be attributed to the geographic isolation caused by the Himalaya-Hengduan Mountains. Two distinct geographic origin centers (Europe and China) were established. Gene tree conflict between nuclear and chloroplast data may result from frequent hybridization among cherry species, as well as the incomplete lineage sorting. The high-altitude regions located at the eastern edge and southern parts of the Himalaya-Hengduan Mountains are likely to have served as glacial refuges for cherries inhabiting in China. Meanwhile, we propose that cultivated Chinese cherries most likely originated in Longmenshan Fault Zone, and subsequently spread throughout four geographic regions, undergoing distinct domestication and divergent selection. This study provides valuable information for the effectively conserving and utilizing cherry resources worldwide.

## Figures and Tables

**Table 1:** Genetic diversity and demographic evolution of the cherry specimens from different groups and lineages assessed using both nuclear ITS and Chloroplast DNA sequences.

Groups and Lineages	Gene Diversity (h)	Nucleotide Diversity (π)	Tajima’s *D* (*p*-Values)	Fu’s *Fs* (*p*-Values)	SSD (*P*_SSD_)	Raggedness Index (*P*_Rag_)
**ITS sequences**						
**Groups**						
CC	0.5378 ± 0.0135	0.0062 ± 0.0051	−1.3853 (0.0620)	−1.1937 (0.3310)	0.0266 (0.0000)	0.2003 (0.0000)
WC	0.5057 ± 0.0311	0.0076 ± 0.0059	−1.2024 (0.1070)	−1.6268 (0.2690)	0.0086 (0.0300)	0.1315 (0.0300)
RC	0.8730 ± 0.0185	0.0447 ± 0.0240	−0.9768 (0.1730)	−0.7611 (0.4900)	0.0127 (0.1600)	0.0213 (0.2200)
EC	0.7381 ± 0.0402	0.0230 ± 0.0136	−1.5179 (0.0320)	−2.1721 (0.2660)	0.0040 (0.0060)	0.0273 (0.0000)
MC	0.8916 ± 0.0137	0.0837 ± 0.0428	0.4441 (0.7490)	5.4135 (0.9290)	0.0164 (0.4100)	0.0201 (0.2900)
**Lineages**						
I (CC + WC + RC)	0.6465 ± 0.0151	0.0189 ± 0.0115	−1.3076 (0.0730)	−14.5974 (0.0060)	0.0353 (0.5600)	0.0901 (0.6700)
II (EC)	0.7141 ± 0.0426	0.0120 ± 0.0081	−1.2283 (0.0850)	−14.7058 (0.0030)	0.0020 (0.7400)	0.0305 (0.9200)
III (RC)	0.9333 ± 0.0620	0.0413 ± 0.0248	−0.7810 (0.2030)	−0.7369 (0.3280)	0.0207 (0.4100)	0.0384 (0.8300)
IV (EC)	0.6667 ± 0.2041	0.0055 ± 0.0062	1.6330 (0.9650)	0.5400 (0.5040)	0.0898 (0.3100)	0.5556 (0.3400)
V (MC)	0.8916 ± 0.0137	0.0837 ± 0.0428	0.4441 (0.7280)	5.4135 (0.9210)	0.0164 (0.3800)	0.0201 (0.1900)
**Chloroplast sequences**						
**Groups**						
CC	0.1440 ± 0.0262	0.0021 ± 0.0024	−2.1891 (0.0000)	−6.1829 (0.0020)	0.0003 (0.3500)	0.5633 (0.7100)
WC	0.6528 ± 0.0375	0.0275 ± 0.0153	0.4693 (0.7110)	−5.8760 (0.0720)	0.0854 (0.1200)	0.1539 (0.0700)
RC	0.9279 ± 0.0135	0.0436 ± 0.0230	−1.1777 (0.0910)	0.6622 (0.6440)	0.0123 (0.0100)	0.0167 (0.0900)
EC	0.6771 ± 0.0444	0.0391 ± 0.0209	−0.1564 (0.5040)	0.6627 (0.6380)	0.0617 (0.3400)	0.1037 (0.3000)
MC	0.8559 ± 0.0216	0.0435 ± 0.0231	0.0161 (0.5700)	0.6622 (0.6460)	0.0308 (0.1000)	0.0371 (0.1600)
**Lineages**						
I (CC + WC + RC)	0.4217 ± 0.0258	0.0167 ± 0.0100	−1.2293 (0.0770)	−26.1216 (0.0000)	0.0523 (0.0900)	0.2725 (0.4000)
II (CC + WC + RC)	0.8023 ± 0.0426	0.0112 ± 0.0074	−1.8758 (0.0080)	−10.5326 (0.0000)	0.0005 (0.8800)	0.0449 (0.5900)
III (EC)	0.8603 ± 0.0554	0.0253 ± 0.0150	−0.4424 (0.3410)	−0.6875 (0.3740)	0.0414 (0.0700)	0.0628 (0.6300)
IV (EC)	0.5404 ± 0.0521	0.0144 ± 0.0090	−0.2552 (0.4640)	1.4514 (0.7710)	0.3687 (0.0000)	0.1874 (1.0000)
V (RC)	0.8629 ± 0.0502	0.0360 ± 0.0199	−0.8824 (0.1980)	−2.7766 (0.1400)	0.0422 (0.0200)	0.0710 (0.0300)
VI (EC)	0.6667 ± 0.2041	0.0046 ± 0.0052	1.6330 (0.9610)	0.5400 (0.4960)	0.0898 (0.3200)	0.5556 (0.3100)
VII (MC)	0.8576 ± 0.0210	0.0432 ± 0.0230	0.0019 (−0.5700)	0.6605 (0.6660)	0.0305 (0.1400)	0.0365 (0.2700)

Note: The lineages I–VII corresponded to the haplotype networks based on ITS and chloroplast sequences (Figure 3 and Figure 4).

**Table 2:** Summary of hierarchical analysis of molecular variance (AMOVA) in various groups of cherry specimens.

Source of Variation	Among Groups (%) ITS/cpDNA	Among Populations within Groups (%) ITS/cpDNA	Within Populations (%) ITS/cpDNA	*F_CT_* ITS/cpDNA	*F_SC_* ITS/cpDNA	*F_ST_* ITS/cpDNA
Five groups	71.97/57.92	18.63/16.04	9.4/26.04	0.7196/0.5792	0.6647/0.3811	0.9060/0.7396
Lineages	84.02/75.06	10.75/10.75	5.24/14.18	0.8402/0.7506	0.6723/0.4312	0.9476/0.8582
Sub-lineages	79.98/82.35	11.40/7.30	8.62/10.35	0.7998/0.8235	0.5692/0.4137	0.9138/0.8965
Three groups (CC, WC, RC)	23.98/30.05	20.61/11.78	55.41/58.17	0.2398/0.3005	0.2712/0.1684	0.4460/0.4183
CC (NEC-C, LFZ-C, YGP-C, QLM-C)	21.53/3.60	22.07/31.05	56.4/65.35	0.2153/0.0360	0.2813/0.3221	0.4360/0.3465
WC (LFZ-W, YGP-W, QLM-W)	26.73/2.69	31.02/19.29	42.25/78.01	0.2674/0.0269	0.4234/0.1983	0.5775/0.2199
RC (LFZ-R, YGP-R, QLM-R)	18.41/18.39	40.67/19.94	40.92/61.67	0.1841/0.1839	0.4985/0.2443	0.5908/0.3833

Note: NEC-C, LFZ-C, YGP-C and QLM-C represented the four geographic subgroups of the cultivated Chinese cherry (group CC). LFZ-W/R, YGP-W/R and QLM-W/R stand for three geographic subgroups of the wild Chinese cherry (WC)/relatives (RC). The codes are same as in in Supplementary Table S1. All *p* value < 0.01.

**Table 3:** Genetic diversity and demographic evolution for different sub-groups and sub-lineages calculated by both nuclear ITS and Chloroplast DNA sequences.

Groups and Lineages	Gene Diversity (h)	Nucleotide Diversity (π)	Tajima’s *D* (*p*-Values)	Fu’s *Fs* (*p*-Values)	SSD (*P*_SSD_)	Raggedness Index (*P*_Rag_)
ITS sequences						
Geographic subgroups of CC, WC and RC						
NEC-C	0.4487 ± 0.0313	0.0037 ± 0.0037	1.5274 (0.9460)	2.0922 (0.7810)	0.0128 (0.0300)	0.2119 (0.0100)
LFZ-C	0.2647 ± 0.0541	0.0054 ± 0.0047	−1.6581 (0.0210)	−0.6743 (0.3650)	0.0044 (0.3800)	0.3250 (0.5500)
YGP-C	0.5342 ± 0.0317	0.0065 ± 0.0053	−1.4573 (0.0480)	−0.3691 (0.4330)	0.0191 (0.0300)	0.1744 (0.0000)
QLM-C	0.5508 ± 0.0701	0.0051 ± 0.0046	0.5080 (0.7810)	0.5710 (0.5820)	0.0224 (0.0400)	0.1840 (0.0700)
LFZ-W	0.5395 ± 0.0321	0.0086 ± 0.0064	−1.1186 (0.1330)	−1.3437 (0.2970)	0.0104 (0.0300)	0.1286 (0.0300)
YGP-W	0.5333 ± 0.1721	0.0044 ± 0.0048	0.8506 (0.8870)	0.6254 (0.4680)	0.0303 (0.3500)	0.2889 (0.5100)
QLM-W	0.2540 ± 0.0953	0.0021 ± 0.0027	−0.0187 (0.3410)	0.4480 (0.3650)	0.2754 (0.1900)	0.3066 (0.1200)
LFZ-R	0.8434 ± 0.0338	0.0249 ± 0.0146	−1.3240 (0.0750)	−2.6787 (0.1540)	0.0037 (0.8400)	0.0141 (0.9600)
YGP-R	0.8536 ± 0.0228	0.0540 ± 0.0286	0.2359 (0.6810)	0.3402 (0.5990)	0.0419 (0.0500)	0.0554 (0.0400)
QLM-R	0.3524 ± 0.1009	0.0109 ± 0.0077	−0.9248 (0.1990)	−0.5961 (0.3790)	0.0331 (0.2200)	0.3015 (0.5200)
Sub-lineages						
i	0.5892 ± 0.0156	0.0117 ± 0.0079	−1.2786 (0.067)	−12.4953 (0.0030)	0.0198 (0.0000)	0.1181 (0.0000)
ii	0.7051 ± 0.0476	0.0201 ± 0.0123	−0.9626 (0.178)	−0.3677 (0.4960)	0.0342 (0.2500)	0.0832 (0.5000)
iii	0.9333 ± 0.0620	0.0413 ± 0.0248	−0.7810 (0.235)	−0.7369 (0.3010)	0.0207 (0.4100)	0.0383(0.8300)
Chloroplast sequences						
Geographic subgroups of CC, WC and RC						
NEC-C	0.0190 ± 0.0186	0.0003 ± 0.0008	−1.3716 (0.0280)	−1.5192 (0.0590)	0.0005 (0.0500)	0.9603 (0.9200)
LFZ-C	0.1823 ± 0.0178	0.0013 ± 0.0018	−2.2798 (0.0020)	0.4346 (0.3970)	0.0005 (0.0400)	0.9646 (0.9300)
YGP-C	0.4059 ± 0.0615	0.0030 ± 0.0030	−0.5075 (0.3220)	−0.8229 (0.3060)	0.0063 (0.1000)	0.1660 (0.3000)
QLM-C	0.1693 ± 0.0841	0.0078 ± 0.0058	−1.4709 (0.0680)	2.0832 (0.8570)	0.0273 (0.1200)	0.7206 (0.6300)
LFZ-W	0.5920 ± 0.0437	0.0248 ± 0.0140	0.3762 (0.7170)	−2.1202 (0.2860)	0.1113 (0.1300)	0.2040 (0.1300)
YGP-W	0.9333 ± 0.1217	0.0276 ± 0.0185	1.7737 (0.9780)	−0.7460 (0.2200)	0.0323 (0.5900)	0.1289 (0.5200)
QLM-W	0.8466 ± 0.0454	0.0390 ± 0.0215	1.9323 (0.9840)	1.4395 (0.7800)	0.0641 (0.0100)	0.1753 (0.0300)
LFZ-R	0.8790 ± 0.0254	0.0277 ± 0.0155	−1.1694 (0.1210)	−4.1397 (0.0770)	0.0127 (0.5400)	0.0299 (0.4800)
YGP-R	0.8176 ± 0.0433	0.0427 ± 0.0227	−0.6262 (0.3020)	−7.1104 (0.0350)	0.0488 (0.0000)	0.0551 (0.0000)
QLM-R	0.9302 ± 0.0213	0.0365 ± 0.0200	0.3232 (0.6920)	−3.8279 (0.0710)	0.0051 (0.8300)	0.0087 (0.9700)
Sub-lineages						
i	0.1590 ± 0.0223	0.0017 ± 0.0022	−1.8953 (0.002)	−21.5495 (0.0000)	0.3400 (0.0014)	0.5549 (0.6400)
ii	0.9168 ± 0.0152	0.0206 ± 0.0120	−1.2586 (0.095)	−3.0942 (0.0800)	0.0083 (0.1700)	0.0309 (0.2700)
iii	0.8965 ± 0.0218	0.0256 ± 0.0145	−1.7462 (0.014)	−1.8477 (0.2300)	0.0054 (0.7900)	0.0135 (0.8900)

Note: NEC-C, LFZ-C, YGP-C and QLM-C represent the four geographic subgroups of cultivated Chinese cherry (group CC). LFZ-W/R, YGP-W/R and QLM-W/R represent three geographic subgroups of wild Chinese cherry (WC)/relatives (RC). The sub-lineages i–iii were identified in the haplotype networks based on ITS and chloroplast sequences (Figure 5 and Figure 6).

## Data Availability

All the sequence data of three chloroplast and one ITS fragments in this paper have been deposited in GenBank database (https://www.ncbi.nlm.nih.gov/nuccore/) with accession numbers KX711711-KX711781.

## Supplementary Materials

The following supporting information can be downloaded at: https://www.mdpi.com/article/10.3390/plants12122258/s1, Figure S1: Geographic locations of the studied cherry accessions. All the samples were collected from five major species types, comprising 330 accessions of 57 local cultivated Chinese cherry populations (group CC), 204 accessions of 13 wild Chinese cherry populations (group WC), 174 accessions of wild relatives from 17 natural populations, ZFRI and KIB (CAS) (group RC), 120 accessions of European cherries (Group EC), and 79 accessions of subgenus *Microcerasus* from 12 populations (group MC). Symbols correspond to the five taxa in the right key, and further details can be found in Supplementary Table S1. The shaded regions and dotted circles with different colors represent four major geographic distribution regions in China: North and East China (NEC), Longmenshan Fault Zones (LFZ), Yun-Gui Plateau (YGP) and Qinling Mountain (QLM). Figure S2: Mismatch distribution of five groups, three main lineages and two sub-lineages for cherry specimens (*Cerasus*) calculated by nuclear ITS sequence. The code name of various resources types, different lineages and sub-lineages are showed in the caption. Figure S3: Mismatch distribution of five groups, four main lineages and three sub-lineages for cherry specimens (*Cerasus*) calculated by chloroplast sequence. The code name of various resources types, different lineages and sub-lineages are showed in the caption. Figure S4: Distance matrix Heat-map of pairwise *Fst* values for cherry specimens (*Cerasus*) calculated by nuclear ITS (below diagonal) and chloroplast DNA (above diagonal) among and five groups (a) and among ten geographic subgroups (b). The value of *Fst* is shown in Heat-map and Significant values are indicated with * *p* < 0.05; ** *p* < 0.01. Table S1: Summary of sample site, geographic origin, taxonomy, sample size, and identified haplotypes for studied cherry resources. Table S2: Information of chloroplast and nuclear DNA fragments. Table S3: Distance matrix of pairwise *Fst* values among five lineages of cherry specimens (*Cerasus*) calculated by nuclear ITS sequence. Table S4: Distance matrix of pairwise *Fst* values among seven lineages of cherry specimens (*Cerasus*) calculated by chloroplast sequence. Table S5: The Demographic and mutation parameters estimated using approximate Bayesian computation for scenario 6. Table S6: The Demographic and mutation parameters estimated using approximate Bayesian computation for scenario 9.
