# Supplementary material for: Phylogeography of 912 Cherry Accessions Insight into Independent Origins of Fruiting Cherries and Domestication Footprints of Cultivated Chinese Cherry (Prunus pseudocerasus Lindl.)
Source: Plants (Basel). 2023 Jun 9;12(12):2258. doi: 10.3390/plants12122258 (PMC10302293; doi:10.3390/plants12122258)
Supplement: Supplementary file 1 [file plants-12-02258-s001.zip › Supplementary Figure S1-S4.pdf]

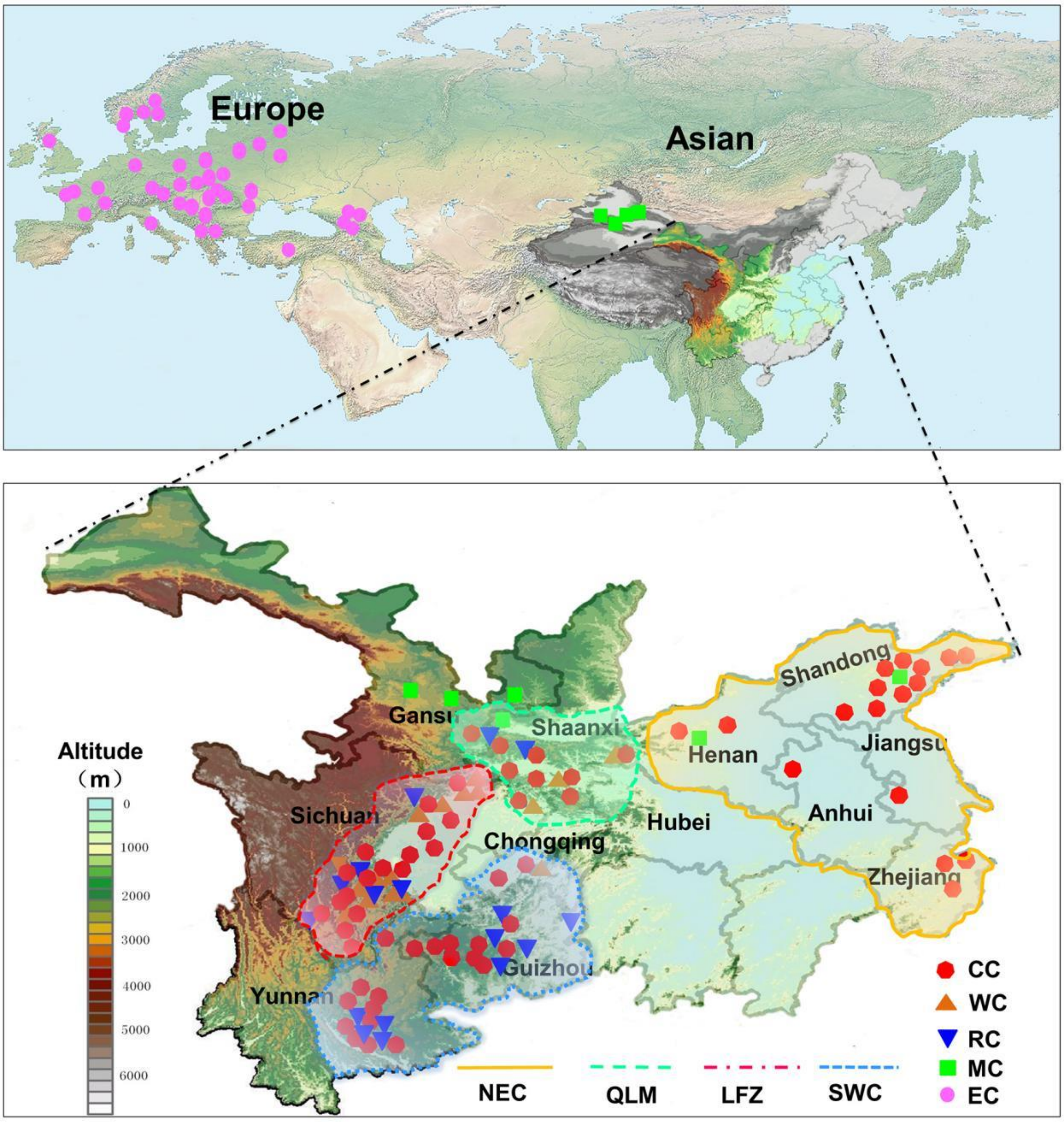

Supplementary Figure S1 Geographic locations of the studied cherry accessions. All the samples were collected from five major species types, comprising 330 accessions of 57 local cultivated Chinese cherry populations (group CC), 204 accessions of 13 wild Chinese cherry populations (group WC), 174 accessions of wild relatives from 17 natural populations, ZFRI and KIB (CAS) (group RC), 120 accessions of European cherries (Group EC), and 79 accessions of subgenus *Microcerasus* from 12 populations (group MC). Symbols correspond to the five taxa in the right key, and further details can be found in Supplementary Table 1. The shaded regions and dotted circles with different colors represent four major geographic distribution regions in China: North and East China (NEC), Longmenshan Fault Zones (LFZ), Yun-Gui Plateau (YGP) and Qinling Mountain (QLM).

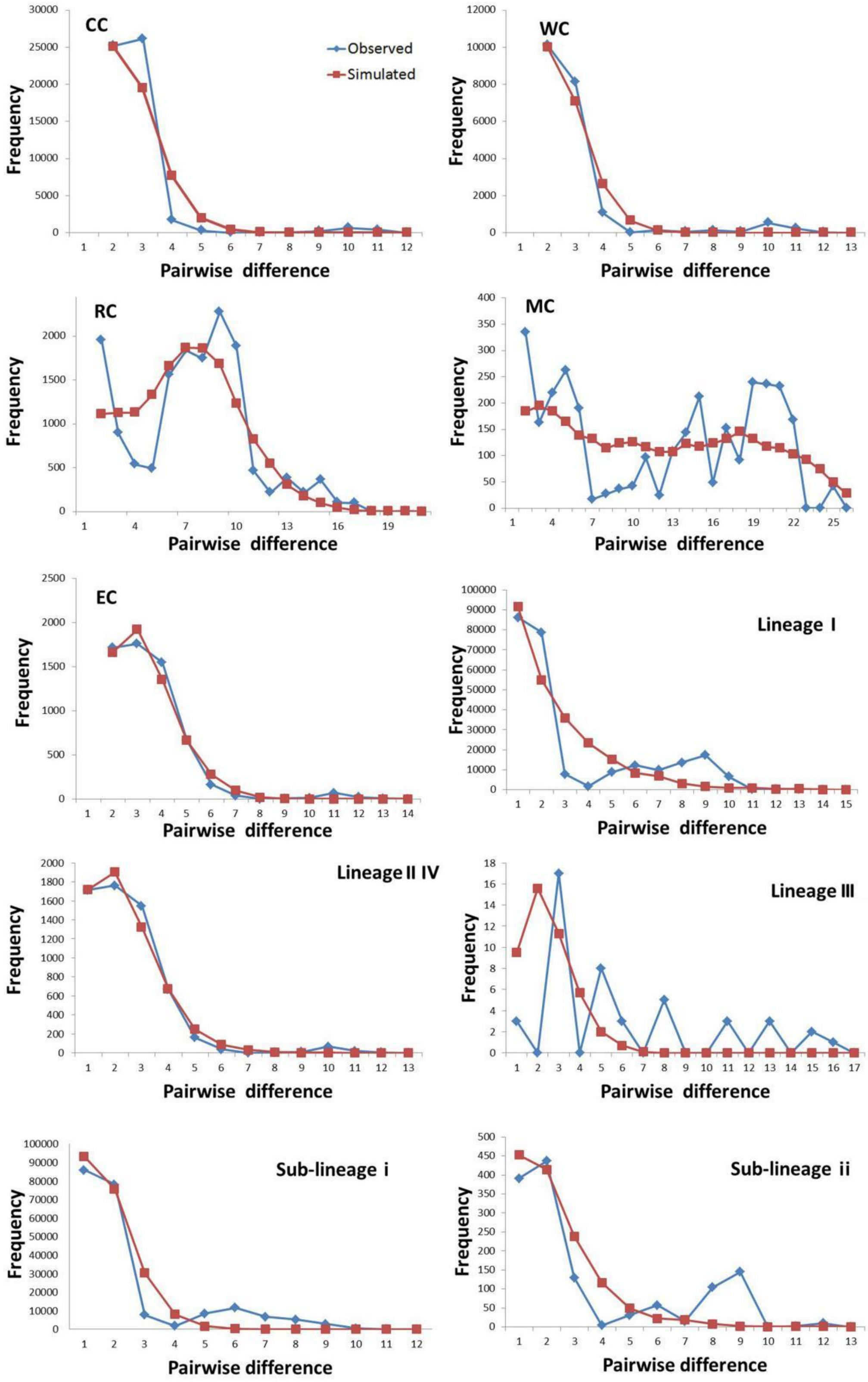

Supplementary Figure S2 Mismatch distribution of five groups, three main lineages and two sub-lineages for cherry specimens (*Cerasus*) calculated by nuclear ITS sequence. The code name of various resources types, different lineages and sub-lineages are showed in the caption.

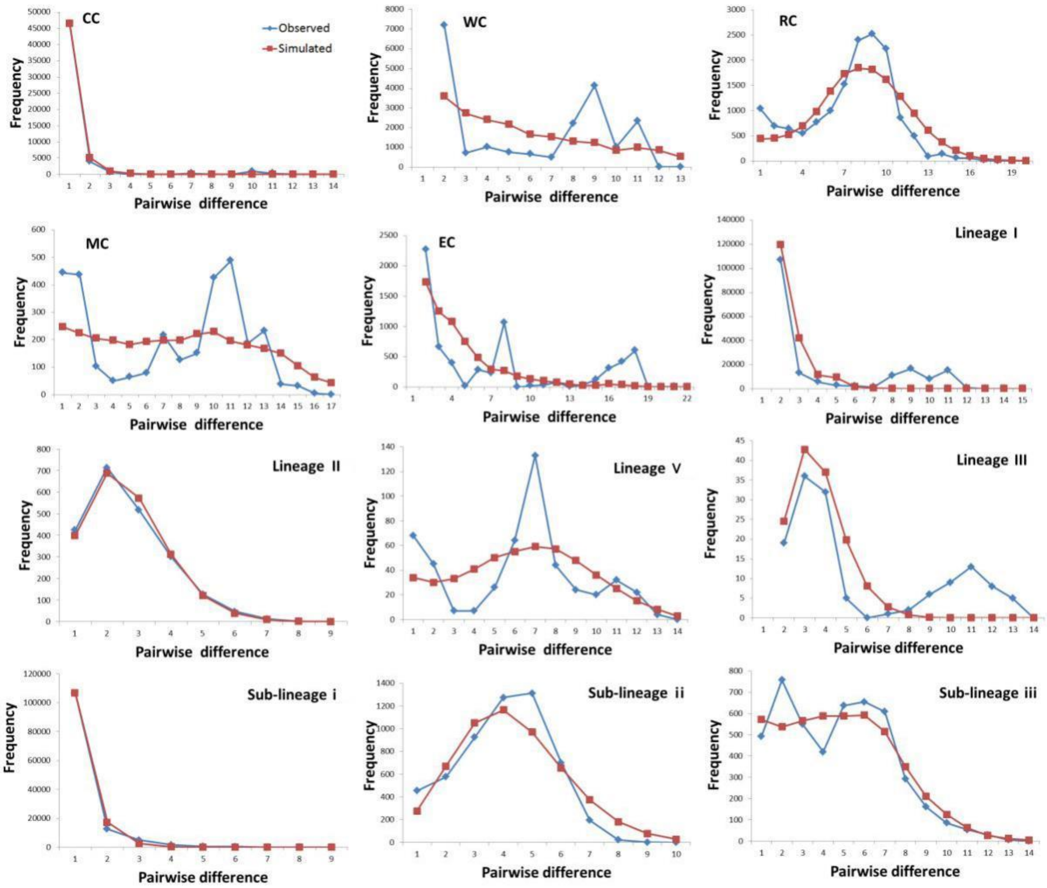

Supplementary Figure S3 Mismatch distribution of five groups, four main lineages and three sub-lineages for cherry specimens (*Cerasus*) calculated by chloroplast sequence. The code name of various resources types, different lineages and sub-lineages are showed in the caption.

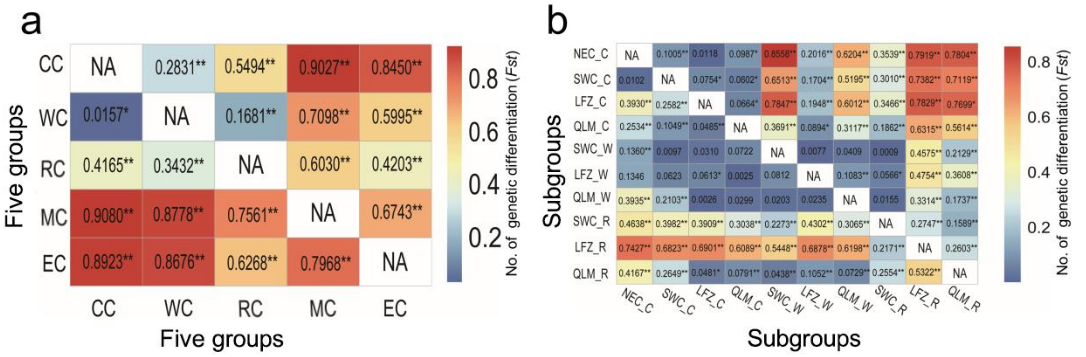

Supplementary Figure S4 Distance matrix Heat-map of pairwise *Fst* values for cherry specimens (*Cerasus*) calculated by nuclear ITS (below diagonal) and chloroplast DNA (above diagonal) among and five groups (a) and among ten geographic subgroups (b). The value of *Fst* are shown in Heat-map and Significant values are indicated with * P < 0.05; **P < 0.01.
